# The UK human T-cell lymphotropic virus cohort: Unique perspectives from a decade of follow up

**DOI:** 10.1186/1742-4690-11-S1-P55

**Published:** 2014-01-07

**Authors:** Katy Davison, Su Brailsford, Patricia Hewitt, Jana Haddow, Graham Taylor

**Affiliations:** 1 NHSBT/PHE Epidemiology Unit, Public Health England, London, UK; 2 Transfusion Medicine, NHS Blood and Transfusion, Charcot Road, Colindale, London. UK; 3 Section of Infectious Diseases, Imperial College, Norfolk Place, London, UK

## 

Human T-cell Lymphotropic virus (HTLV) prevalence is low in the UK, but higher in individuals originating from endemic countries. Anti-HTLV screening of all blood donations was introduced in 2002 to reduce the risk of transfusion transmission. The UK HTLV Cohort (UHC) was established for longterm follow up of people affected by HTLV. Recruitment by clinicians at blood centres and specialist clinics began in July 2003. Participants are asked to complete a baseline self completion questionnaire (SCQ) about their health and HTLV risk factors, flagged in central registries for cancer or death, and followed up about their health and well being every 2-3 years. Recruiting clinicians report participant’s clinical state at diagnosis. By March 2013, a total of 161 HTLV positive individuals had consented to take part: 89 of these were blood donors. Most were female (77%), of non-white ethnicity (64%) with a mean age of 49 years. Most (83%) positive participants were asymptomatic for HTLV at registration and eight (4%) participants died during follow-up. Baseline questionnaires were received from 160 (85%). Follow up was done in 2003, 2007 and 2010, the fourth is underway. Once complete, approximately 1,000 person years of follow up will be analyzed, factors associated with symptoms will be investigated and an assessment of the progression of HTLV related disease among the UHC will be presented. Information from the UHC, obtained from a decade of follow up, will provide a unique perspective since it is the only cohort of its kind in Europe.

